# Cell Trafficking at the Intersection of the Tumor–Immune Compartments

**DOI:** 10.1146/annurev-bioeng-110320-110749

**Published:** 2022-04-06

**Authors:** Wenxuan Du, Praful Nair, Adrian Johnston, Pei-Hsun Wu, Denis Wirtz

**Affiliations:** 1 Institute for NanoBiotechnology Department of Chemical and Biomolecular Engineering, and Johns Hopkins Physical Sciences Oncology Center, Johns Hopkins University, Baltimore, Maryland, USA; 2 Department of Oncology, Department of Pathology, and Sidney Kimmel Comprehensive Cancer Center, Johns Hopkins University School of Medicine, Baltimore, Maryland, USA

**Keywords:** random migration, chemotaxis, immunotherapy, tumor microenvironment

## Abstract

Migration is an essential cellular process that regulates human organ development and homeostasis as well as disease initiation and progression. In cancer, immune and tumor cell migration is strongly associated with immune cell infiltration, immune escape, and tumor cell metastasis, which ultimately account for more than 90% of cancer deaths. The biophysics and molecular regulation of the migration of cancer and immune cells have been extensively studied separately. However, accumulating evidence indicates that, in the tumor microenvironment, the motilities of immune and cancer cells are highly interdependent via secreted factors such as cytokines and chemokines. Tumor and immune cells constantly express these soluble factors, which produce a tightly intertwined regulatory network for these cells’ respective migration. A mechanistic understanding of the reciprocal regulation of soluble factor–mediated cell migration can provide critical information for the development of new biomarkers of tumor progression and of tumor response to immuno-oncological treatments. We review the biophysical and biomolecular basis for the migration of immune and tumor cells and their associated reciprocal regulatory network. We also describe ongoing attempts to translate this knowledge into the clinic.

## INTRODUCTION

Cell trafficking plays a central role in critical physiological processes that drive tumor progression, particularly in cancer metastasis and in immune cell infiltration and escape. In metastasis, cancer cell migration through the stromal matrix drives the spread of cancer cells from a primary tumor site to distant organs (1, 2). In immune tumor infiltration, the immune response to tumor cells depends critically on the recruitment of immune cells to cancer sites, and this process fundamentally relies on immune cell migration (2, 3). Misregulated migration of immune cells can result in the failure of their response to cancer cells and lead to immune evasion and ineffective immunotherapy (4, 5). Because most immunotherapies require direct cell–cell contact, an understanding of migration is required to therapeutically enhance infiltration of antitumor immune cells while blocking the recruitment of protumor immune cells. Tumor infiltration of immune cells is highly regulated by both cancer cells and immune cells, as cancer cells are capable of immunoediting the microenvironment to enhance protumor immune cell localization and repel antitumor immune cells, which in turn allow certain protumor immune cells to enhance the ability of cancer cells to metastasize. Tumor infiltration by immune cells is a critical yet mostly unmet clinical need, as more than 90% of cancer deaths are caused by metastatic disease.

It is well established that focal adhesions, intracellular polarization, actin filament assembly, and myosin-mediated contractility (6, 7), regulated via Rho GTPases, form the nexus of various signaling pathways that regulate cell migration, including Ras, phosphatidylinositol 3-kinase (PI3K), and focal adhesion kinase (FAK) (8-10). Recent studies have shown that soluble factors secreted by cancer and immune cells play a significant role in regulating one another’s migration (4,11-13). In the tumor microenvironment (TME), cancer and immune cells are generally close to one another, and their interactions through secretory factors including chemokines and cytokines compose a complex network for mutual regulation of migration, which has a profound impact on tumor initiation and progression. Extracellular vesicles (EVs), which are nonsoluble factors secreted by tumor and immune cells, have also emerged as prominent regulators of immune response (14, 15) and cell migration (16-19) in the TME. However, in this review we focus mainly on soluble factors as mediators of migration.

A detailed mechanistic understanding of the role of soluble factors produced by both immune and cancer cells and the activation of downstream pathways resulting in both immune and cancer cell migratory modulations into, within, and out of TMEs is critical to our understanding of cancer progression (12, 13). An in-depth understanding of the interplay between cancer and immune cells in regulating their motility could lead to a category of treatments that target cancer and/or immune migration, since there are, to date, no drugs approved by the US Food and Drug Administration (FDA) that aim to directly modulate cancer and immune cell migration. These potential drugs may be stand-alone, such as those that block or reduce metastasis, or complementary to current treatments, such as those that may increase antitumor immune cell tumor infiltration to enhance current immunotherapies whose effectiveness is limited by low immune cell tumor infiltration (20-24).

In this review, we first categorize cell migration into two distinctive patterns, chemotaxis and random migration, based on parameters like directionality, persistence, and speed, which work as a combination of “steering wheel” and “engine” to modulate the entire migration process. After introducing currently available in vitro and in vivo methods, we summarize the reciprocal regulation of cell migration between immune and cancer cells by comprehensively reviewing upstream intercellular molecular cross talk mediated by soluble factors and corresponding receptors. At the end, we describe relevant clinical trials to provide insights into potential therapies targeting cancer and immune cell migration.

## MODES OF CELL MIGRATION

**Figure 1:**
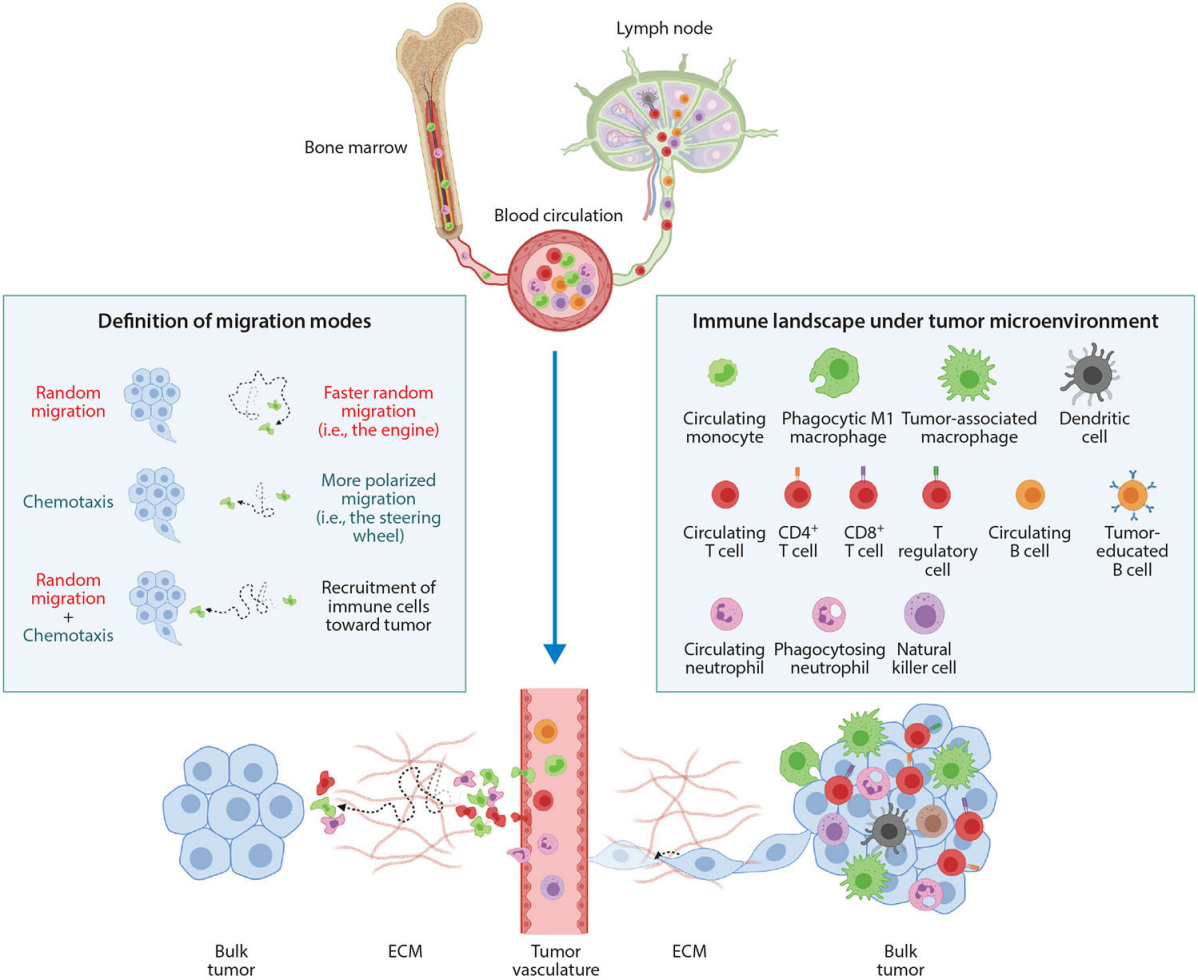
Chemotaxis versus random migration of immune cells in the tumor microenvironment. Chemotaxis is defined as directionally biased cell migration along a chemical gradient, while basal migration is nondirectional (random). Cell migration in a chemotactic gradient does not necessarily result in straight-line trajectories, as cell movement is typically a combination of pure chemotaxis and basal random migration. In particular, immune cells that are actively recruited by tumors exhibit a combination of enhanced basal migration and biased migration. Under a panoramic view of immune cell infiltration, mature immune cells are released to peripheral blood circulation and will accumulate outside tumor vasculature after extravasation. Via a combination of chemotaxis and basal migration, infiltration of immune cells occurs in a relatively short time and consequently creates a hot tumor microenvironment, which initiates further tumor cell invasion and metastasis. In contrast, the motility of cancer cells is typically far slower than the motility of immune cells. Abbreviation: ECM, extracellular matrix. Figure adapted from images created with BioRender.com.

**Figure 2:**
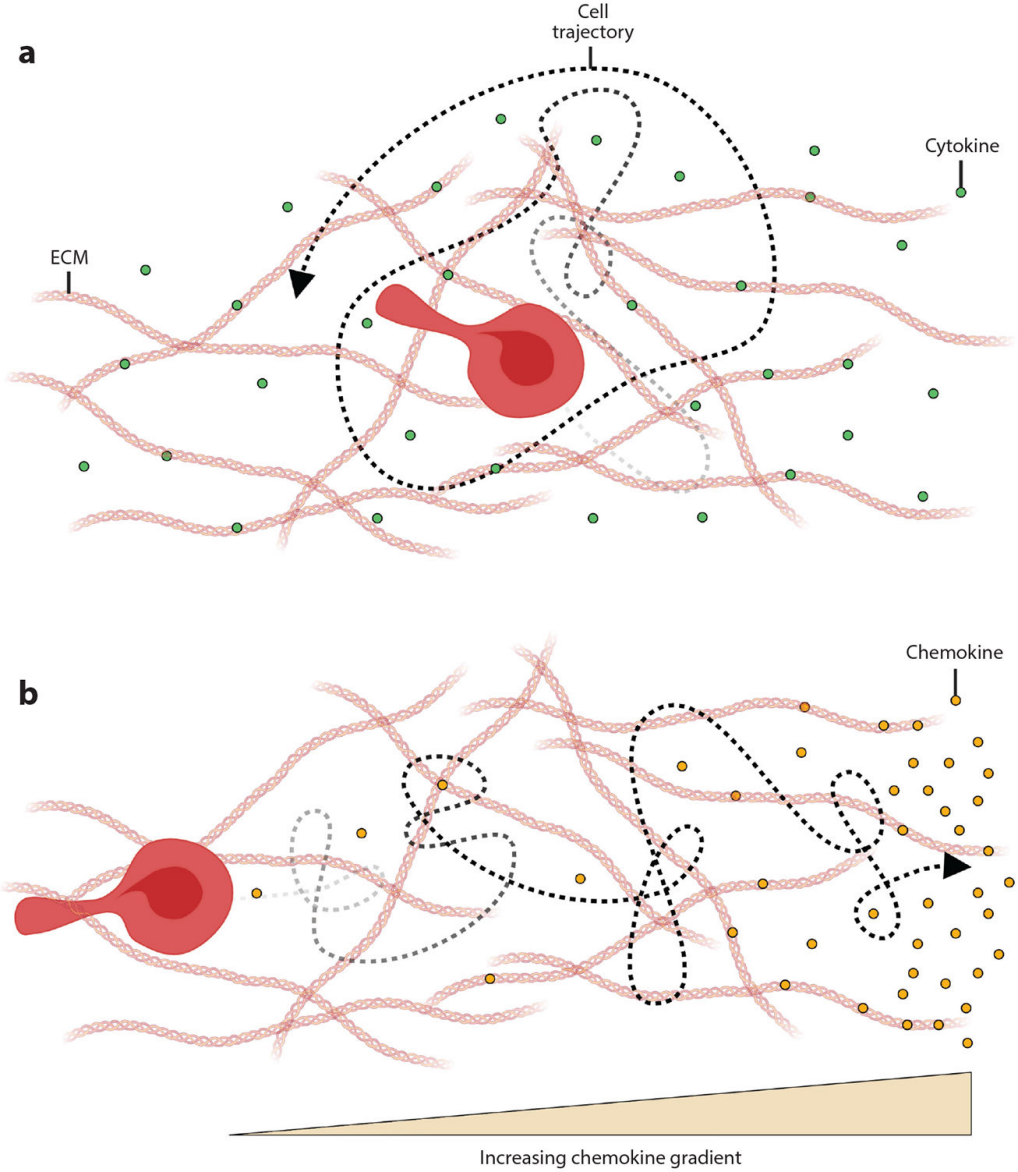
The role of (*a*) cytokines and (*b*) chemokines in cell movement. (*a*) Cytokines are a class of soluble factors that regulate a variety of cellular functions, such as proliferation and differentiation. (*b*) Chemokines are a class of cytokines that promote directional cell migration (i.e., chemotaxis). Cells migrate in the direction of increasing chemokine gradients. Abbreviation: ECM, extracellular matrix. Figure adapted from images created with BioRender.com.

Because chemotaxis and basal random migration—the two main modes of migration of immune and cancer cells—are regulated by completely different molecular pathways, it is critical to distinguish these processes. Chemotaxis consists of biased, directional migration along chemical gradients produced by neighboring cells (Figure 1) and is the basis for infiltration of tumors by immune cells (13). Cancer cells can modulate immune cell recruitment to select for protumor immune cells, including myeloid-derived suppressor cells (MDSCs) and T regulatory cells (Tregs) (4), which then inhibit immunosurveillance (25, 26). Chemotaxis is also exploited by cancer cells, especially during metastasis, as soluble factors and EVs can promote tumor cell migration toward invasive margins and recruitment to secondary tumor sites (4, 27). In contrast to chemotaxis, basal migration consists of unbiased (random, nondirectional) movement that occurs in the absence of chemical gradients (Figure 1). Basal cell migration typically occurs within the TME—especially in the stromal matrix—where the concentrations of soluble factors tend to be more constant compared with the interfacial space between blood vessels and tumors, where gradients of soluble factors are steeper. Key among these soluble factors are cytokines, which are secreted proteins that regulate a variety of cellular functions, such as proliferation and differentiation, and drive cell migration (28, 29). A subclass of cytokines, called chemokines, drive migration via chemotaxis (30, 31) (Figure 2). Chemotactic and basal migration of immune cells within the TME describe the extent to which immune cells explore the tumor to elicit anti- or protumor functions (Figure 1).

## QUANTITATIVE CELL MIGRATION ASSAYS IN VITRO AND IN VIVO

**Figure 3:**
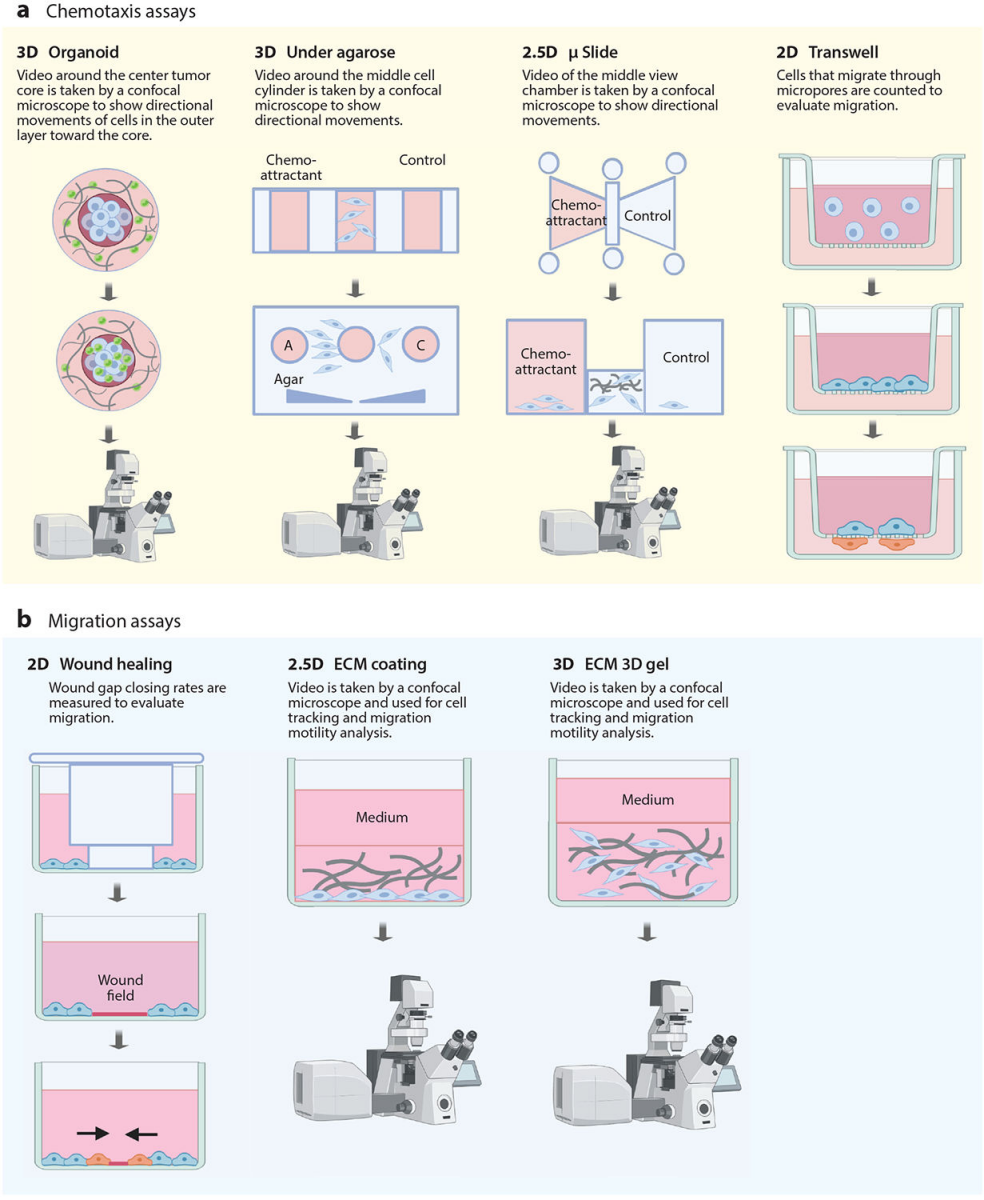
Standard assays used to study (*a*) cell chemotaxis and (*b*) basal migration in vitro. (*a*) Chemotaxis is typically studied in vitro by placing immune or cancer cells between a well containing chemoattractant molecules in medium on one side and medium on the opposing side. (*b*) Basal migration is studied in two dimensions (2D) on plastic and in three dimensions (3D) in gels constructed using extracellular matrix (ECM) proteins, such as collagen I and fibronectin. Time-lapse microscopy is used to visualize cell migration and extract motility parameters such as the speed and persistence of individual migratory cells, while cell counting is used for Transwell^®^ assays to extract bulk parameters of invasion. We denote 2.5D as a setting where cells are allowed to adhere to an ECM-coated dish and ECM is deposited on the apical surface of the cells. These cells are not fully embedded into the ECM as in 3D. Figure adapted from images created with BioRender.com.

**Figure 4:**
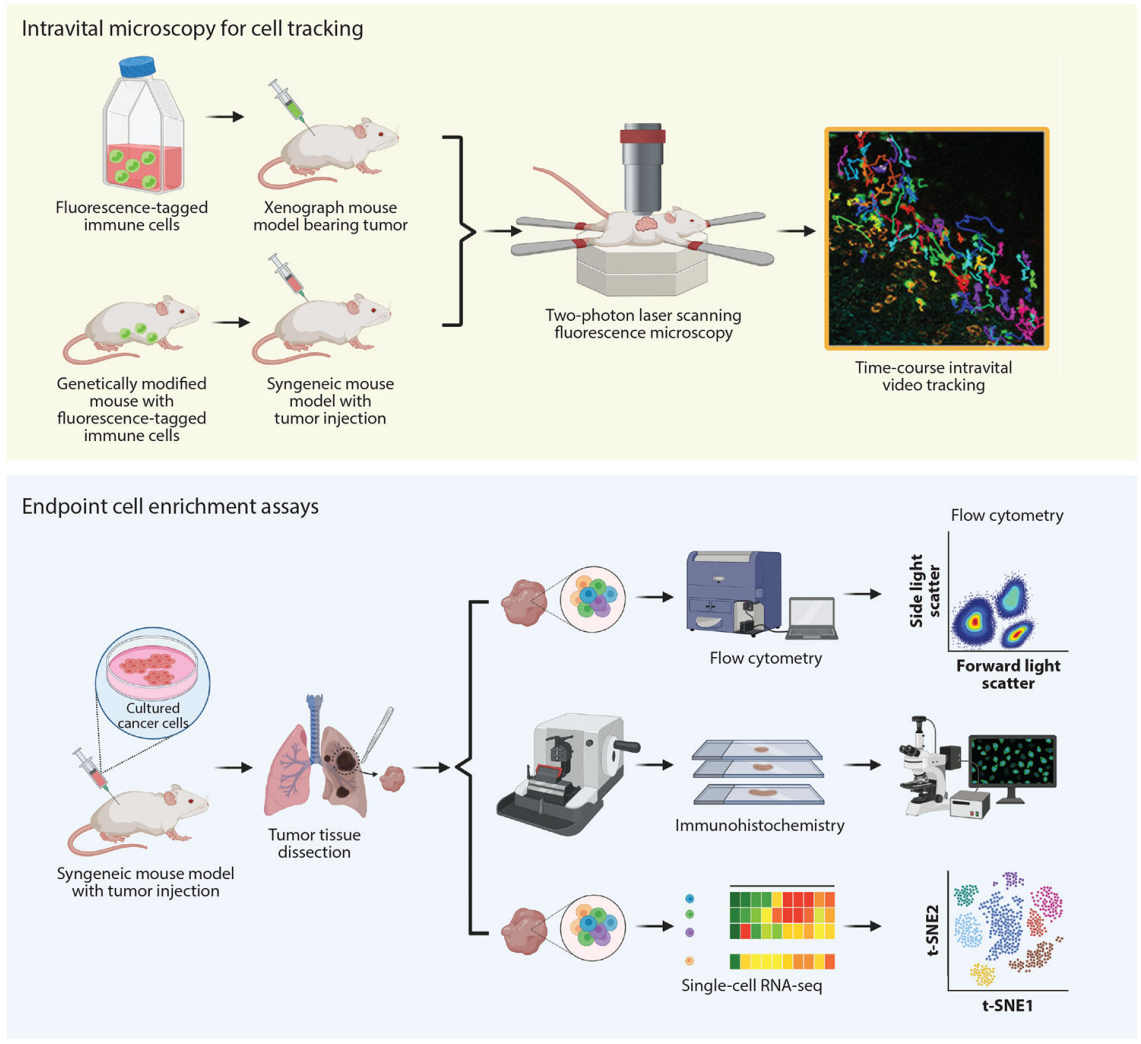
Standard assays to study cell enrichment and migration in vivo. Due to the complexity of setting up fluorescence-tagged mouse models suitable for intravital microscopy, under most circumstances endpoint assays are adopted to study cell enrichment at primary or secondary tumor sites. Unlike in vitro assays, which can distinguish between chemotaxis and random migration, endpoint cell enrichment assays can focus on the presence of cells only at specific time points and sites, which usually arise not only from chemotaxis and random migration but also from proliferation. Syngeneic mouse models with intact immune systems are injected with cultured cancer cells. After tumor establishment and progression, tumor tissues are dissected and subsequently stained with various cell markers for immunohistochemistry, flow cytometry, or single-cell RNA sequencing (RNA-seq). Immune cells found in the tumor or stroma are viewed as enriched or recruited. Intravital microscopy (e.g., two-photon laser scanning fluorescence microscopy) can overcome the shortcomings of endpoint cell enrichment assays by tracking individual cells in real time. Cancer cells are injected into either xenograft mice transferred with fluorescence-tagged immune cells or syngeneic mice that are genetically modified with fluorescence-tagged immune cells. Time-course intravital video tracking can then be carried out to study real-time cell migration in vivo. Abbreviation: t-SNE, t-distributed stochastic neighbor embedding. Image for time-course intravital video tracking from Reference 50. Figure adapted from images created with BioRender.com.

**Figure 5:**
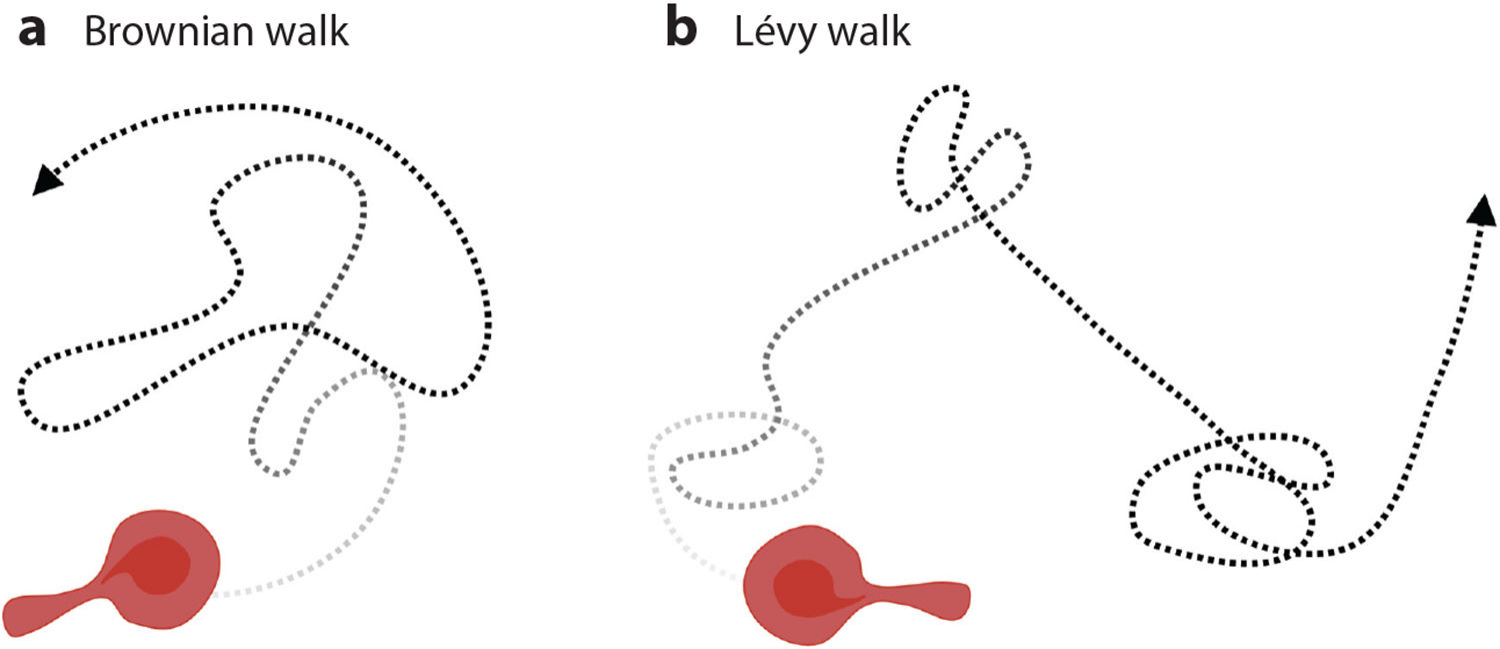
Trajectories of (*a*) Brownian walk and (*b*) Lévy walk. Cell movements under Brownian walk follow a Gaussian distribution, while cell movements under Lévy walk are composed of small movements interspaced with long exploratory excursions. Figure adapted from images created with BioRender.com.

The distinct molecular mechanisms of chemotaxis and basal migration of immune and cancer cells have been identified mainly in vitro using a plethora of quantitative bioengineering assays (Figure 3). Coupled with the ease of cellular manipulation, these assays can readily quantify cell migration both in two dimensions (32-39) and in more physiological three-dimensional settings (40-43), both in bulk and at the single-cell level. In contrast, direct assessment of cell chemotaxis and migration in vivo has proven to be more challenging. Most in vivo assays for migration consist essentially of a black box, as they largely rely on endpoints (44-48) and are unable to distinguish chemotaxis from random migration (Figure 4). This is problematic because the enrichment of immune cells in a tumor could be due to immune cell proliferation at the tumor site, for instance, rather than enhanced infiltration and colonization. In vitro assays and intravital microscopy in vivo (49-52), which rely on time-lapse microscopy to monitor real-time single-cell movements, are the only assays in which basal migration and chemotaxis can be distinguished. Only in these scenarios can motility—and associated parameters such as spatial and temporal directional persistence, mean-squared displacement, distributions of cell movements, and diffusivity—be properly defined and measured (see the sidebars) (Figure 5).

Time-lapse microscopy has been the central tool to study cell migration in vitro and in vivo. The development of accurate and automated computational methods for cell tracking using time-lapse videos has long been challenging. Traditionally, cell tracking is achieved by manually locating and tracking cells in each frame, often aided by open-source tools such as ImageJ/FIJI via MtrackJ (53) and TrackMate (54) plugins. Although a trained researcher can accurately identify and track cells, these workflows are not well suited for a large number of images and can be subject to bias. Pattern-matching algorithms and contour evolution methods have been established to computationally track the same cells from frame to frame and derive their trajectories (55-58). In addition, the use of image processing approaches to locate the centroids of individual cells in each image through cell segmentation or image filtering as well as probabilistic frameworks to establish temporal associations between cells are effective computational cell tracking approaches (59-61). The open-source tool CellProfiler has been employed for segmentation-based cell tracking (62, 63). Recent advances in artificial intelligence (AI), particularly in cellular segmentation, could be integrated into segmentation-based cell tracking to further improve its robustness and accuracy. AI tools such as Cellpose (64) and SegNet (65) have superior accuracy and robustness in segmenting cells compared with classical image processing–based cell segmentation methods. With advances in microscopic imaging hardware and AI-integrated cell tracking analytics, time-lapse videos containing cells can be acquired and analyzed in a high-throughput manner and can allow for the study of cell motility at a systems level.

Motility parameters, such as speed and persistence time, are in most cases collected for a relatively small number of cells and are presented in the form of average values, which fail to properly take into account cellular heterogeneity (66, 67). Single-cell transcriptional profiling methods are widely applied to depict molecular portraits of collective responses of heterogeneous cell populations to external stimuli (68-70). Parallel advances in high-throughput single-cell motility phenotyping platforms coupled with powerful data science approaches will help analyze enormous sets of cell tracking data (66, 71, 72), providing mechanistic frameworks to fully couple dynamic cell trafficking patterns with molecular signatures and functional behaviors.

## IMMUNE CELL CHEMOTAXIS REGULATED BY CANCER CELLS

The infiltration of immune cells into a tumor is a prerequisite for antitumor immunity, whereby subsets of immune cells—such as CD8^+^ T cells and natural killer (NK) cells—elicit cytolytic activity through cell–cell interactions with tumor cells (26). Mechanisms that control tumor infiltration by immune cells are key to the effectiveness of immunotherapy. In this section, we critically review recently uncovered molecular mechanisms of directed (chemotactic) migration of specific immune cells—macrophages, neutrophils, T cells, and dendritic cells (DCs)—mediated by cancer cells.

### Macrophages

Tumor-associated macrophages (TAMs) play a key role in tumor-associated immunosuppression (77). Macrophages can induce either inflammation or immunosuppression through their polarization into an M1 or M2 phenotype, respectively (77). The molecular mechanisms of how tumors regulate the enrichment of M2-polarized TAMs are actively being studied. Hypoxia, the depletion of oxygen observed in the cores of numerous tumors, is known to affect immune cells through metabolism and function (78, 79) and induces TAMs to help shape the TME into an immunosuppressive environment by the release of soluble factors. For example, TAMs in hypoxic environments release higher levels of interleukin (IL)-6 and IL-10, which can increase programmed cell death protein 1 (PD-1) expression on T cells, as well as higher levels of CCL17 and CCL22, which induce protumor Tregs to chemotax to tumors (80, 81). But recent evidence suggests a different role for hypoxia in modulating cancer cells, not only immune cells directly, to elicit downstream immunosuppression. Under hypoxic conditions, melanoma cells secrete exosomes that contain elevated levels of the chemoattractants CCL2 and colony-stimulating factor 1 (82), which are also thought to polarize macrophages into the M2 anti-inflammatory phenotype (83, 84). Transwell^®^ assays (Figure 3) show enhanced macrophage chemotaxis toward exosomes derived from hypoxic melanoma cells compared with exosomes from melanoma cells under normoxic conditions; these hypoxic exosomes push macrophages into a M2 phenotype in a metabolic-dependent manner (82). In fact, in vivo mouse model endpoint enrichment assays (Figure 3) showed that higher numbers of M2-like cells are present in hypoxic melanoma tumors than in normoxic melanoma tumors (82). Therefore, hypoxic tumors seem to promote macrophage chemotaxis into the TME (41) to induce their differentiation into protumor M2 phenotypes (83, 84), leading to an immunosuppressive TME. M2 macrophages then exacerbate the immunosuppressive TME by secreting CCL22 and CCL17 to attract another class of protumor immune cells, Tregs (80, 81).

### Neutrophils

Similar to that of macrophages, the role of neutrophils in cancer biology is complex and can both promote and suppress tumor progression (85, 86). Supporting their role in promoting tumor progression, recent evidence has shown that neutrophils in tumors of patients with triple-negative breast cancer (TNBC) correlate with decreased tumor growth (87). This neutrophil inhibition may be regulated by the chemokines CXCL1 and IL-8 released by TNBC cells. Metabotropic glutamate receptor 1 (mGluR1) is overexpressed on TNBC cells, and this overexpression leads to downregulation of TNBC cell–released CXCL1 and IL-8 (87-89). Conditioned medium of TNBC cells with silenced or overexpressed *GRM1*, the mGluR1-encoding gene, effectively promotes or inhibits, respectively, neutrophil chemotaxis (87). Pan-neutrophil infiltration is likely not hampered by TNBC cells; rather, antitumor neutrophils may be selected against while protumor neutrophils are recruited (90), providing additional credence to the possibility that TNBC cells attempt to shape the immune landscape in the TME through soluble factors.

One way in which neutrophils may be preferentially recruited to secondary tumors of TNBC cells that have metastasized is through the C3a/C3a receptor axis. A TNBC syngeneic mouse model bearing TNBC cells that had metastasized to the liver showed that liver-metastatic TNBC cells secrete a higher level of C3a (90), a soluble factor of the complement system, than do lung-metastatic TNBC cells. Indeed, preferential neutrophil recruitment is exhibited in response to liver-metastatic cells as opposed to lung-metastatic cells (90). Protumor neutrophils express higher levels of the C3a receptor than do antitumor neutrophils, which accounts for protumor neutrophils’ preferential infiltration (90).

### T Cells

T cells are among the most widely studied immune cell types in immuno-oncology, partly because of their potential therapeutic efficacy against a host of tumor types. An outstanding question is how to increase CD8^+^ T cell tumor infiltration, as it is thought that infiltration of this T cell subtype into tumor cores, rather than mere accumulation along tumor margins, leads to improved patient clinical outcomes (91). Understanding the mechanisms by which the TME hampers cytotoxic T cell infiltration may lead to improved T cell immunotherapies.

Gliomas are one of the cancer types for which developing immunotherapies have proven challenging. The gain-of-function *IDH1* mutation is one of the most frequently observed mutations in glioma (92). Patients harboring this mutation present reduced tumor-infiltrating cytotoxic T cells. Gliomas in orthotopic syngeneic glioma mouse models bearing the *IDH1* mutation compared with gliomas in mice wild type (WT) for *IDH1* also have reduced tumor-infiltrating cytotoxic T cells, as well as reduced chemokine CXCL10 expression and reduced STAT1-positive cells. Therefore, CXCL10 production is hampered in *IDH1*-mutated glioma cells in a STAT1-dependent manner, seemingly correlating with reduced tumor infiltration. The supernatants of in vitro cultured *IDH1*-mutant glioma cells are thus suspected to contain less CXCL10 compared with those of *IDH1*-WT glioma cells; this hypothesis is validated by a Boyden chamber assay showing that CD8^+^ T cell chemotaxis is roughly 3.5-fold less when T cells are chemotaxing toward the supernatant of *IDH1*-mutant glioma cells (93). Antibody-mediated blockade of CXCR3, CXCL10’s cognate receptor, also reduces CD8^+^ T cell chemotaxis toward the supernatant of CXCL10-containing *IDH1*-WT glioma cells by roughly 3.5-fold (93). Hence, it can be inferred that reduced CXCL10 secretion may be heavily involved in *IDH1*-mutant glioma cells’ strategy to hamper CD8^+^ T cell chemotaxis. Interestingly, the presence of CXCR3 as well as another chemoattractant receptor, BLT1, on T cell membranes in a syngeneic melanoma mouse model may be a requisite for T cell infiltration into tumor cores (94). Knocking out these receptors in T cells abrogates their presence in tumor cores, while their presence at tumor peripheries does not seem to change in comparison to control T cells (94).

Ovarian cancer is another cancer for which immunotherapy development has posed challenges. Ovarian cancer cells can epigenetically silence CCL5 (95), a known T cell chemoattractant (96-98). Multispectral imaging of human ovarian tumor sections has revealed an association between CD8^+^ T cell accumulation and CCL5 (95). Chemotaxis assays in Transwells have demonstrated that blocking the cognate receptor of CCL5, CCR5, hampers T cell chemotaxis toward ovarian TME conditioned medium (95).

### Dendritic Cells

Different subtypes of DCs exist (99, 100). Tumor infiltration of antitumor DC subsets, such as CD103^+^ DCs (101), may lead to better cancer prognosis because of their function of antigen presentation to T cells (102). Yet, certain subsets of DCs, such as pre-DCs, can be immunosuppressive (103). Transwell assays (Figure 3) have shown that ovarian epithelial carcinoma cells can recruit pre-DCs exhibiting immunosuppressive phenotypes, and stromal cell–derived factor 1 (SDF-1) is the key secretory factor through which ovarian cancer cells induce DC chemotaxis (103). Melanoma is among the cancer types for which immunotherapies have proven successful, although there is room for improvement, given that some patients have low antitumor immune cell infiltration (104). Melanoma patients exhibiting active β-catenin signaling have worse clinical outcomes (105). This discrepancy may be due in part to decreased secretion of chemokine CCL4 by melanoma cells arising from active β-catenin signaling, resulting in hampered CD103^+^ DC tumor infiltration and impeded CD103^+^ DC chemotaxis (106). Whether the reduction in CCL4 secretion works alone or in tandem with the depletion of other soluble factors is hard to say, since conditioned medium from melanoma cells with active β-catenin signaling is depleted of other soluble factors as well (106).

## CANCER CELL CHEMOTAXIS REGULATED BY IMMUNE CELLS

Remarkably little research has focused on cancer cell chemotaxis toward immune cells. However, a recent study demonstrated that T cells at invasive margins of colorectal cancer tumors at liver-metastatic sites secrete chemokine CCL5. Invasion chamber assays reveal that colorectal cancer cells chemotax toward CCL5 (107). Under agarose, Transwell, and μ-slide assays (Figure 3), either using ex vivo conditioned medium of T cells from the invasive margin or knocking out *CCL5* in T cells in a colorectal cancer mouse model bearing liver metastases and monitoring cancer cell migration to the invasive margin through intravital microscopy (Figure 4) would demonstrate in vitro whether CCL5 produced by T cells promotes cancer cell chemotaxis to invasive margins. Apart from immune cells, tumor-associated lymphatic endothelial cells promote chemotaxis through lymphatic vessels (108, 109) and to premetastatic lymph nodes (109, 110). Yet, whether and how lymph node–resident immune cells specifically contribute to observed cancer cell chemotaxis remain unknown.

Because chemoattractants are among the mechanisms that regulate immune cell tumor infiltration, we note that certain secreted molecules—including IL-6 and IL-8—are dependent on cancer cell density in the tumor (43). Therefore, future studies might reveal how immune cell chemotaxis fits within the framework of the origins of cancer when the first few cancerous cells arise. Such studies could address how, before forming tumors, small numbers of cancerous cells repel antitumor immune cells and recruit tumor-promoting immune cells to help form a microenvironment favorable to tumor formation. More research is necessary to understand how immune cells promote metastasis at distant sites through cancer cell chemoattractants. Future research could determine whether tissue-resident immune cells in distant organs secrete soluble factors that prompt cancer cells at the primary site to chemotax toward the organs in which those tissue-resident immune cells reside. It could also show whether, once recruited to distant organs, these small numbers of cancer cells recruit and repel certain immune cell types to promote secondary tumor formation.

## REGULATION OF RANDOM MIGRATION OF CANCER CELLS BY IMMUNE CELLS

**Figure 6:**
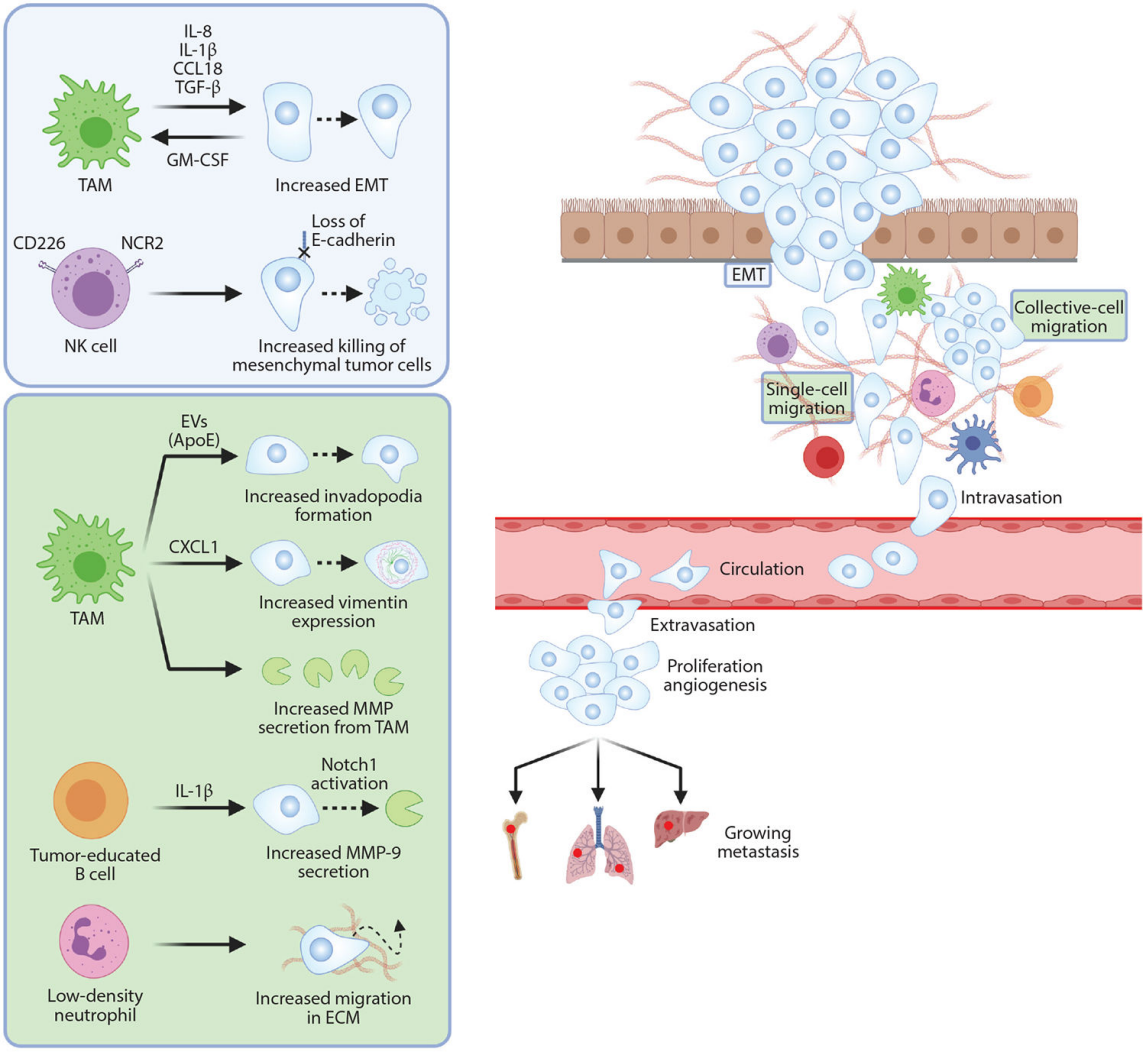
The role of immune cells in cancer cell migration. (*Left*) The metastatic cascade. (*Right*) Effects on cancer cells’ epithelial-to-mesenchymal transition (EMT) and random migration steps are summarized in the blue and green boxes, respectively. The cytokines interleukin (IL)-8, IL-1β, CCL18, and transforming growth factor (TGF)-β are produced by tumor-associated macrophages (TAMs) as boosters of cancer cells’ EMT. During EMT, a positive feedback loop is completed via cancer-secreted granulocyte-macrophage colony-stimulating factor (GM-CSF), which works as a TAM differentiation activator. Meanwhile, CD226/NCR2-positive natural killer (NK) cells more effectively eliminate cancer cells undergoing EMT by recognizing their loss of E-cadherin, an NK cell inhibitory receptor. During cancer cell random migration in the stromal extracellular matrix (ECM), the chemokine CXCL1 and exosomes containing apolipoprotein E (ApoE) derived from TAMs can increase invadopodia formation in cancer cells. In addition, elevated secretion of matrix metalloproteinases (MMPs) from TAMs (together with cancer cells, themselves induced by IL-1β from tumor-educated B cells) promote cancer cell migration. Low-density neutrophils are also protumoral as a result of their capability to increase cancer cell migration, which in turn facilitates metastasis. Abbreviation: EV, extracellular vesicle. Figure adapted from images created with BioRender.com.

Immune cells recruited to the invasive front and core of a tumor play a critical role not only in the proliferation and death of the constitutive cancer cells but also in the cells’ migration. Cancer cell invasion through the basement membrane and migration into the stromal matrix are key drivers of tumor progression and metastasis. In the remainder of this review, we describe the molecular mechanisms that different types of immune cells exploit to modulate cancer cell migration. Below, we systematically review the role of immune cells in cancer cell migration (summarized in Figure 6).

### Macrophages

Cancer cells acquire basal migration capacity and initiate metastasis through the epithelial-to-mesenchymal transition (EMT), a phenotypic switch from a homeostatic state to cell invasion and migration. The loss of the cell membrane molecule E-cadherin during EMT induces a dual loss of intercellular adhesion and apical–basal polarity, resulting in a mesenchymal motile phenotype that allows cancer cells to stretch along the collagen scaffold of the stromal matrix (111, 112). A connection between EMT and stromal immune infiltration was originally established by observing colocalization of TAMs with hepatocellular carcinoma invasive hot spots (77). Transforming growth factor (TGF)-β has long been known to be a potent inducer of EMT through SMAD-mediated activation (111). The detection of TAM-derived TGF-β confirmed the enhancing effect of TAMs on EMT processes (111). A positive feedback loop of the cytokine granulocyte-macrophage colony-stimulating factor (GM-CSF) and the chemokine CCL18 has also been demonstrated: CCL18 secreted by TAMs primes cancer cells into a mesenchymal-like phenotype, and in turn, these cancer cells upregulate the expression of GM-CSF as a differentiation activator of monocytes into TAMs (113). More recently, other soluble factors secreted by TAMs—including IL-8 and IL-1β—have been found to boost EMT (114).

Multiple TAM-derived genes and cytokines have been associated with poor clinical prognosis. Macrophages play a pivotal part in cancer migration by promoting cancer cell migration and remodeling the extracellular matrix (ECM) (115). Conditioned medium from TAMs applied to cancer cells in the Transwell invasion assay and the wound-healing assay promotes cancer cell migration motility (77, 113, 114). S100A8/S100A9 are upregulated in colon and lung carcinoma cells after treatment with TAM conditioned medium, leading to increased cancer cell migration (116). The chemokine CXCL1 (117) and exosome-containing apolipoprotein E (118), derived from TAMs, promote cancer cell migration. In addition, Notch1/Mena^INV^ initiate invadopodium formation in cancer cells in a macrophage-dependent manner (119). In combination with the increased motility of cancer cells, TAMs themselves are also capable of secreting ECM-degrading enzymes, including cathepsins and matrix metalloproteinases (MMPs) (120).

### B Cells

Unlike in TAMs, whether B cells have a protumoral or antitumoral effect remains unclear. Here we focus mainly on tumor-educated B cells (TEBs) in the TME and their interactions with cancer cells to promote basal cell migration. A recent study showed that IL-1β secreted by TEBs promotes renal carcinoma cell migration by potentiating hypoxia-inducible factor (HIF)-2α expression. HIF-2α increases the expression of *DLL4* at the transcriptional level by binding directly to site 3 of the *DLL4* promoter region, which then activates Notch1 signals, causing downstream secretion of MMP-9 for increased cancer cell migration (121).

### Natural Killer Cells

NK cells play a crucial role in the immunosurveillance of cancer cells. NK cells do not directly mitigate the migration capacity of tumor cells; instead, they very effectively target invasive cancer cells with a high migratory potential (122). E-cadherin has been identified as an NK cell inhibitory receptor. Loss of E-cadherin during EMT transition makes the resulting migrating cancer cells susceptible to recognition and elimination by specific subtypes of NK cells, specifically those cells that overexpress NCR2 (natural cytotoxicity-triggering receptor 2) and CD226 (123). IL-15 has a potent cytoprotective effect on NK cells because it leads to the development of NK cells that express the T-bet family member eomesodermin, resulting in more efficient killing of invasive cancer cells (124).

### Neutrophils

Neutrophils are the most abundant leukocyte subpopulation circulating in peripheral blood, so the chemotaxis of neutrophils toward cancer-associated inflammation has been extensively studied. However, the ability of these cells to tune tumor cell migration has been much less explored. The phenotypic diversity of neutrophils was discovered in a murine breast cancer model, which demonstrated distinctive roles of high-density neutrophils as antitumoral and low-density neutrophils as protumoral (86). Further characterization has shown that the response of low-density neutrophils to granulocyte colony-stimulating factor secreted by cancers is a signal of recruitment, which facilitates metastasis of 4T1 breast cancer cells and CT26 colorectal cancer cells in syngeneic mouse models (125). In a recent study, the formation of superenhancer regions with aberrantly high transcription factor binding in various C-X-C-type chemokines’ genes in inflammatory ccRCC (clear cell renal cell carcinoma) cells was identified as the inducer of production of the corresponding massive C-X-C-type chemokines, including CXCL1, CXCL5, and CXCL8, for neutrophil recruitment. Targeted bromodomain and extraterminal motif inhibitor treatment in vivo counterbalanced neutrophil-dependent cancer migration and metastasis (126). Unfortunately, direct in vitro and in vivo assessments of cancer cell migration are lacking.

### T Cells

Remarkably little is known about the potential effect of T cells on cancer cell migration. Applying conditioned medium harvested from cytotoxic CD8^+^ T cells or immunosuppressive Tregs on cancer cells seems to cause differences only in proliferation, not in invasion capacity or migration potential. However, indirect cross talk between T cells and other immune cells in TMEs may occur. In particular, infiltrating CD8^+^ and CD4^+^ T cells in a colorectal cancer model can deliver chemotactic cytokine CCL5 (107), which promoted cancer cell invasion and migration through repolarization of macrophages into tumor-associated phenotypes in a simple collagen-coated Transwell assay.

## REGULATION OF IMMUNE CELL MIGRATION INDUCED BY CANCER CELLS

Coordinated migrations are essential for immune cells to patrol the body for pathogens and inflammation. Some immune cells, such as neutrophils and effector T cells, are short-lived and extravasate out of circulation only in the presence of danger signals (127-129). Other cells, including innate lymphoid cells, macrophages, DCs, and NK cells (130), as well as the more recently discovered resident memory T (Trm) cells (131), can adapt to local tissue niches and reside in nonlymphoid organs (127). In most cases, leukocyte extravasation involves tethering, rolling, adhesion, crawling, and transmigrating through endothelial barriers (128).

NK cells and Trm cells can undergo homeostatic proliferation in the event of stress (127, 130, 131). There might also be a significant progenitor population as an emergency reservoir, akin to the myelocyte “lazy pool” for rapid neutrophil replenishment (132). Tissue-resident macrophages and DCs can also self-renew without the input of circulatory progenitor pools (133, 134). Distinguishing between immune cell enrichment through chemoattraction and local proliferation is necessary for tailoring targeted cancer therapies. Because both chemotaxis to inflammatory sites and emigration to draining lymph nodes require the activation of migratory machinery, we believe that intrinsic migration is important for achieving immunosurveillance and is at least partially responsible for invasion. The vast majority of the research described below stems from in vitro studies and would benefit greatly from validation in vivo.

### Modulation of Immune Cell Migration via Proteins Secreted by Cancer Cells

Tumor necrosis factor (TNF)-related apoptosis–inducing ligand (TRAIL) induces apoptosis in cancer cells (135-137). TRAIL also decreases the motility of Jurkat cells (a T cell line) by decreasing intracellular calcium, leading to depolymerization of actin filaments (138). Additionally, exposure to TRAIL reduces the adhesion of Jurkat cells to the ECM molecule laminin, further decreasing cell migration in laminin-rich ECM.

SDF-1 plays a critical role in cancer cell metastasis (139) and is associated with a poor prognosis in cervical cancer (140). Additionally, SDF-1 has been implicated in the induction of T cell migration (141,142). SDF-1 regulates the motility of Jurkat cells by activating RhoA and RhoC, proteins involved in actin filament assembly (143). In addition to Rho proteins, the Wiskott–Aldrich syndrome protein (WASP) induces cytoskeletal rearrangements that promote cell migration. WASP lies downstream of a member of the Rho family of GTPases, Cdc42 (144), and the interaction between Cdc42 and WASP is essential for SDF-1-induced primary T cell chemotaxis (145). SDF-1α induces phosphorylation of WASP and FAK, along with a few other cytoskeletal proteins (146). SDF-1-induced cell migration has been attributed to reactive oxygen species (ROS) (143) and nitric oxide (NO) (147) signaling. SDF-1-induced actin filament rearrangement is abrogated by treating cells with ROS and NO synthase inhibitors, establishing the downstream role of ROS and NO in Jurkat cell migration.

Phosphatase and tensin homolog (PTEN) is an important tumor suppressor that checks the activity of PI3K, a prominent oncogene that influences cell growth, metabolism, and motility (148). PI3K promotes cytoskeletal reorganization and metastasis of cancer cells (148-150). In Jurkat cells, PTEN plays an important role in regulating actin polymerization, hence controlling Jurkat cell migration (151). Increased PTEN expression leads to CXCL12-induced actin polymerization and increased F-actin levels, as measured by increased phalloidin incorporation in cells expressing PTEN. Interestingly, PTEN-mediated cell migration has little effect on the directionality of cell migration. PI3K activation is essential in CXCL12-induced Jurkat cell migration (152). Src kinases, which regulate activation of the PI3K pathway (153, 154), are crucial as well, and treatment of Jurkat cells with a PI3K inhibitor reduced their migration.

The vast majority of the studies reported above have been performed on immortalized cell lines such as Jurkat cells. Validation of these findings in primary cells is lacking.

### Modulation of Immune Cell Migration via Extracellular Matrix Remodeling

Cytokines produced during inflammation elicit a wide range of behaviors in immune cells, including proliferation (155-157), differentiation (155, 158, 159), and activation (1, 156). A prominent cytokine secreted by cancer cells is TNF-α (1, 160, 161). TNF-α can influence the production of fibronectin (162, 163) and also binds to it, impeding T cell migration (164). The migration of T cells through fibronectin-enriched ECM depends on integrins, such as integrin α_V_,which binds to fibronectin and is overexpressed in inflamed ECM (163).

Cancer cells also produce a variety of proteases that digest ECM molecules (165, 166), including MMPs, cathepsin B, and urokinase-type plasminogen activator (165). The degradation of ECM creates physical pathways in the stromal matrix for cancer cells to metastasize. However, this process could also facilitate the trafficking of immune cells to the tumor. MMPs are among the most-studied proteases, and production of MMPs such as MMP-9 in cancers promotes metastasis and angiogenesis (166-168). Macrophages produce MMP-9 following exposure to a specific laminin α5 peptide (169). This process leads to chemotaxis and infiltration of macrophages and neutrophils in tumors. Notably, the overexpression of laminin and MMP-9 has also been reported in cancer cells (165, 166, 170).

### Proteins Secreted by Cancer Cells That Modulate Immune Cell Proliferation, Differentiation, and Activation

Cytokines and inflammatory factors produced by cancer cells can affect immune cells by altering their proliferation, as well as their behavior toward cancer cells, via changes in their differentiation and activation status. These changes make the infiltrated immune cells promote tumor progression rather than oppose it. Cancer cell secretions can regulate macrophage polarization and convert an antitumor macrophage to a protumor macrophage (77, 171). Conditioned media from Lewis lung carcinoma cells induced macrophage activation (172). Versican, an ECM proteoglycan present in the conditioned medium, is responsible for this effect. Versican induces TLR-2-mediated macrophage activation, leading to secretion of TNF-α. TNF-α is important for cancer cell extravasation and intravasation during metastasis. Versican also binds to hyaluronan, another abundant ECM material in tumors, and these ECM components increase cancer cell migration. Together, these processes enhance Lewis lung carcinoma metastasis, providing an elegant example of how cancer cells can tune immune cells to their own benefit.

In addition to TNF-α, another important cytokine in tumor progression is TGF-β (1, 161). TGF-β plays an important but paradoxical role in tumor growth and metastasis by suppressing tumor growth yet promoting metastasis (173, 174). In keeping with this paradoxical theme, conflicting reports of the effects of TGF-β on the immune system have been published. TGF-β has been reported to suppress immunosurveillance by inhibiting T cell proliferation and activation (175, 176) but also to increase the immunosuppressive M2-type macrophage population and to suppress cytotoxic NK cells (177).

However, TGF-β promotes the differentiation of specific T cell subtypes (such as Th17, Th19, and Trm), improving immunosurveillance (176). TGF-β also leads to the recruitment of tumor-associated neutrophils (TANs) (178). TGF-β blockade decreases this population of TANs while enhancing the influx of cytotoxic TANs, thus increasing antitumor response.

**Figure 7:**
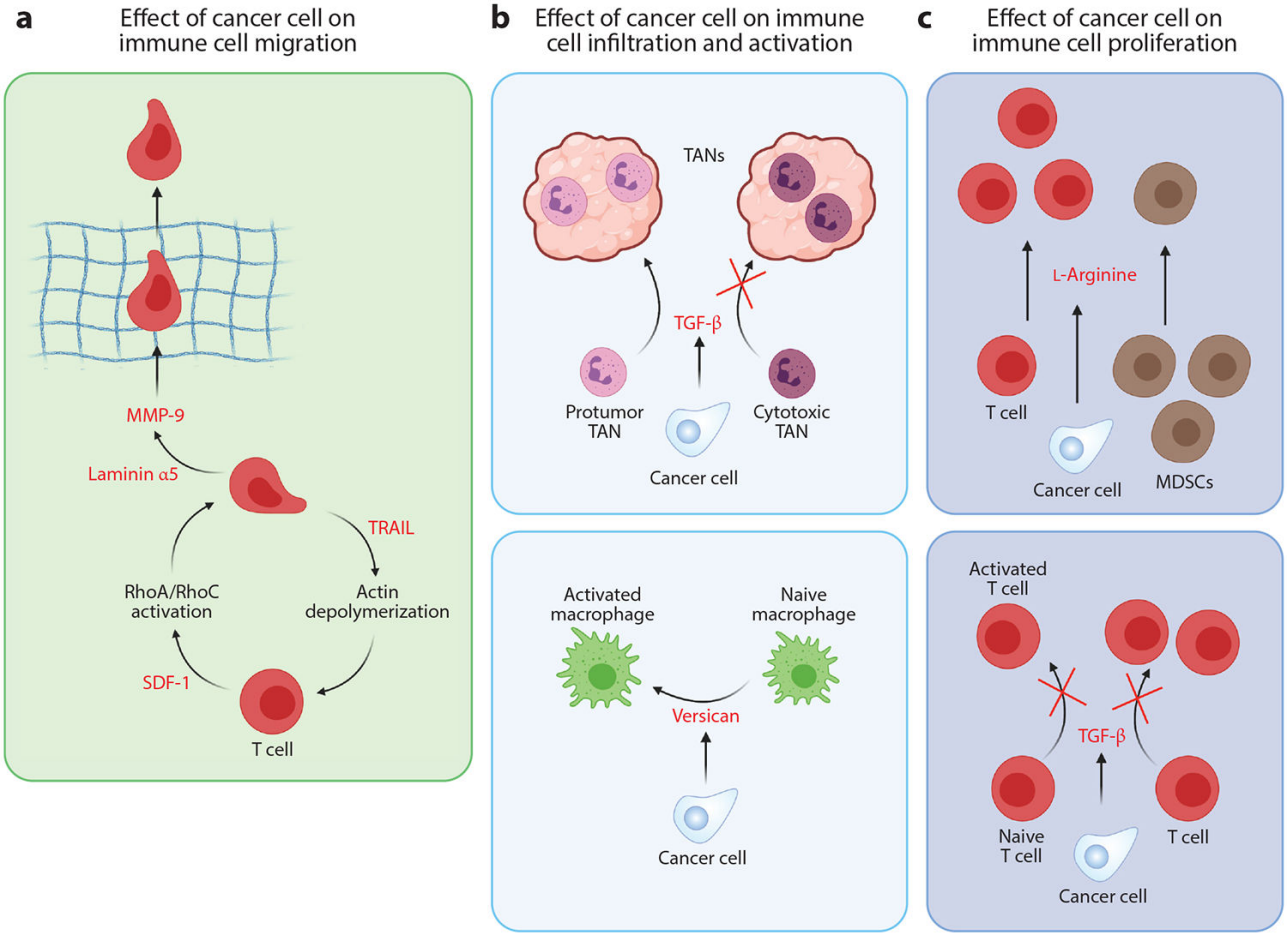
Effect of soluble molecules secreted by cancer cells on immune cell function. (*a*) Cancer cells can affect T cell migration by producing factors that alter their actomyosin contractility and enhance their ability to degrade the extracellular matrix (ECM). Stromal cell–derived factor 1 (SDF-1) induces RhoA activation, while tumor necrosis factor–related apoptosis–inducing ligand (TRAIL) promotes actin filament disassembly. Laminin α5 leads to the production of matrix metalloproteinase 9 (MMP-9), which is essential for ECM degradation. (*b,c*) Additionally, cancer cells can influence the infiltration, proliferation, and activation of immune cells such as neutrophils, macrophages, and T cells. Transforming growth factor (TGF)-β secreted by cancer cells assists the infiltration of protumor tumor-associated neutrophils (TANs) while preventing the infiltration of cytotoxic TANs and conversion of naive T cells to activated T cells. Versican secreted by cancer cells aids in the activation of macrophages. Finally, l-arginine promotes proliferation of T cells but suppresses that of myeloid-derived suppressor cells (MDSCs). Figure adapted from images created with BioRender.com.

Still other factors may play similar roles in both cancer cells and immune cells. One such factor is the amino acid arginine. Cancer cells typically feature altered metabolism, and some types of cancers show high dependency on arginine. Cancers that are arginine auxotrophic (i.e., cannot synthesize arginine) are particularly vulnerable and have been considered for arginine deprivation therapy to reduce tumor growth (179-181). l-Arginine has been described as important for T cell metabolism, and its deprivation could lead to cell cycle arrest and reduced T cell numbers (182). Systemic administration of l-arginine prolongs the survival of immunocompetent mice bearing breast tumors (183). Administration of l-arginine increases the population of T cells in vivo in 4T1 tumor–bearing BALB/c mice while reducing the numbers of immunosuppressive MDSCs. These conflicting reports reveal a fine cancer type–dependent balance between (*a*) targeting the cancer cells and causing tumor regression and (*b*) targeting the immune system and aiding tumor growth. Figure 7 summarizes the effect of cancer cells on immune cell migration.

## CLINICAL RELEVANCE

In addition to the proteins and small molecules discussed above, numerous other factors influence the function of immune cells. Many of them are secreted by cancer cells and are involved in modulating immune–cancer cell interactions or tumor surveillance. In this section, we highlight proteins with prognostic value that are secreted by cancer cells and play a vital role in regulating immune cell function.

### Drugs That Modulate Immune Cell Migration

Though FDA-approved drugs directly targeting immune cell migration and chemotaxis in cancer implications are few to nonexistent, both ongoing and completed clinical trials have aimed to modulate immune cell infiltration into tumors. Plerixafor is a small-molecule inhibitor targeting CXCR4, currently approved to enhance hematopoietic stem cell transplants in the blood vessel from the bone marrow by blocking the interaction of CXCR4 (receptor) on hematopoietic stem cells with SDF-1 (ligand, chemokine) secreted by stromal cells (184). A recently completed clinical trial showed that treatment with plerixafor in advanced pancreatic, ovarian, and colorectal cancer patients increases the number of T cells and NK cells at the tumor sites (Table 1, NCT02179970) (185). Additional clinical trials are assessing plerixafor in other oncological applications, such as its combination with a PD-1 inhibitor (Table 1, NCT04177810).

Apatinib is a small-molecule inhibitor of tyrosine protein kinase on vascular endothelial growth factor receptor 2 that hampers angiogenesis (186, 187). It is being studied in multiple clinical trials, including in combination with camrelizumab for the improved tumor infiltration of lymphocytes and blockade of immunosuppressive myeloid cells (188-190) (Table 1, NCT04523662). Sitagliptin is a drug used to treat type 2 diabetes, specifically to inhibit dipeptidyl peptidase 4 (DPP-4) (191). Because of its role in diminishing biologically active CXCL10 production and improving infiltration of CXCR3^+^ T cells and NK cells into tumors (192), DPP-4 is currently being studied in a clinical trial for patients with hepatocellular carcinoma (Table 1, NCT02650427).

A preclinical mouse model has shown that the nonsteroidal anti-inflammatory drug celecoxib enhances T cell recruitment to tumors by blocking the immunosuppressive constitutive expression of indoleamine 2,3-dioxygenase 1 driven by cyclooxygenase-2 expression (193). As a result, celecoxib is the subject of an ongoing clinical trial for patients with endometrial carcinoma (Table 1, NCT03896113).

Interferon-α_2a_ linked to polyethylene glycol (peginterferon alfa-2a) (47) is an immunosuppressive drug used to treat hepatitis B and C (194-196). A clinical trial to determine the effect of peginterferon alfa-2a on T cell recruitment to tumors in colon cancer patients is underway (Table 1, NCT04798612).

### Potential Biomarkers for Immune Cell Migration

Clinical trials focusing on stimulating and priming the immune system against tumors have attracted increasing interest. However, clinical trials that aim primarily to directly modulate immune cell migration and their trafficking into tumors are sparse. We present a look into clinical trials involving potential biomarkers that could also influence immune cell migration. These trials could form a stepping-stone to studies of potential correlation between these biomarkers and immune cell infiltration into tumors.

### CCL3

Lower levels of CCL3 [also known as macrophage inflammatory protein 1α (MIP-1α)] have been associated with a poor prognosis and increased risk of some types of cancers (197,198). Consistent with these observations, CCL3 enhances antitumor effects by recruiting and priming various types of immune cells, including T cells, B cells, NK cells, and DCs (199-202). Because recruitment of immune cells is an important function of CCL3, CCL3 has been hypothesized to play an important role in the migration of these recruited cells. This hypothesis was tested using Jurkat cells. CCL3/MIP-1α is required for the transendothelial migration of these cells (203). This migratory ability is linked to the expression of adhesion proteins VCAM-1 (vascular cell adhesion molecule 1) and ICAM-1 (intercellular adhesion molecule 1) by MIP-1α. Table 2 summarizes clinical trials that have examined CCL3/MIP-1α as a potential biomarker.

### C-Reactive Protein

Elevated levels of C-reactive protein (CRP) indicate poor prognosis in a variety of cancers, including colorectal, lung, breast, and ovarian cancers (204, 205). CRP is produced in the liver by hepatocytes in response to IL-6 (206). Cancer cells can produce IL-6 (which stimulates the production of CRP) or, in some cases, CRP (207). While a direct role for cancer cell–induced CRP in immune cell proliferation has not been found, several reports suggest that CRP may play a role in T cell proliferation. In one study, CRP reduced the yield of bone marrow–derived DCs in vitro, which in turn reduced T cell proliferation (208). In another study, CRP inhibited the proliferation and function of activated CD4^+^ and CD8^+^ T cells (209). CRP can also lead to the production of monocyte chemoattractant protein 1, which affects the migration and infiltration of monocytes and macrophages (203). Finally, CRP can lead to increased production of IL-6 and IL-8, which play a critical role in cancer cell metastasis (106). CRP levels are routinely assessed as a marker of inflammation; Table 3 summarizes ongoing clinical trials that examine CRP as an outcome measure.

## FUTURE DIRECTIONS

Given that secreted cytokines and chemokines affect cell migration and chemotaxis, coculture studies consisting of cancer cells and one or more types of immune cells are needed to further study the bidirectional regulation of cell motility. Future studies would also require the inclusion of other cell types present in the TME, such as fibroblasts and endothelial cells.

Clinical trials are needed to gain more insight into the potential prognostic role of cytokines and chemokines and to determine whether these proteins can serve as biomarkers of one or more types of cancer. Once a possible prognostic role is established, the underlying mechanism would be of interest, particularly if it involves influencing the motility of cancer cells or immune cells. Advantages of establishing such biomarkers would include rapid and cost-effective cancer diagnosis and management.

Chemotaxis and random migration must be studied with different assays because they are different modes of migration. Chemotaxis incorporates directionality, whereas random migration is nondirectional. Therefore, the assays that define chemotaxis are not interchangeable with the assays that define random migration, improving our understanding of the distinct molecular networks that regulate both.

Three-dimensional systems better mimic the physiological environment of a tumor than commonly used two-dimensional culture dishes.

No cell–cell contact between immune cells and tumor cells will occur without chemotaxis and basal migration of immune cells, and no chemotaxis will occur without basal migration. Understanding both will lead to better clinical outcomes for current cell–cell contact immunotherapies, such as chimeric antigen receptor T cells and checkpoint inhibitors. Armed with such knowledge, researchers in the field will be able to design combination therapies with checkpoint inhibitors where cancer escape/evasion can be reversed by blocking the migration and/or chemotaxis of immunosuppressive immune cell subtypes, such as monocytes, Tregs, and Th2 cells, and enhancing the infiltration of tumor-suppressive immune cell types, such as cytotoxic CD8^+^ T cells, Th1 cells, and DCs, by enhancing their basal migration and chemotaxis toward TMEs. For instance, blocking the IL-6 receptor on monocytes abrogates its basal migration, thus inhibiting monocytes from even being able to chemotax to TMEs and infiltrate tumors. This effectively keeps the TAMs and MDSCs they differentiate into out of the TME.

Plotting average values of migration parameters fails to reveal cells’ dynamic phenotypes. Advances achieved in the bioengineering field on high-throughput single-cell motility phenotyping platforms should be applied to the mechanistic discovery of cancer biology to provide brand-new perspectives.

## Figures and Tables

**Table 1:** Ongoing and completed clinical trials of drugs directly targeting immune cell recruitment to tumors

Clinical trial identifier	Immune cell type	Purpose of study
NCT02179970	T cells and NK cells	Safety of continuous intravenous administration of plerixafor in patients with advanced pancreatic, ovarian, and colorectal cancers
NCT04523662	Multiple immune cell types	Effectiveness and safety of camrelizumab combined with apatinib mesylate and radiotherapy in the treatment of advanced liver cancer
NCT02650427	T cells and NK cells	Safety of a 3-week sitagliptin treatment in HCC patients undergoing liver resection
NCT03896113	T cells	Neoadjuvant celecoxib in newly diagnosed patients with endometrial carcinoma
NCT04798612	T cells	Effect of low-dose interferon-α_2a_ on perioperative immune suppression

Abbreviations: HCC, hepatocellular carcinoma; NK, natural killer.

**Table 2:** Ongoing clinical trials focusing on CCL3/MIP-1α as a biomarker

Clinical trial identifier	Cancer type	Purpose of study
NCT00319748	Breast, ovarian, endometrial, and cervical	Effect of a TLR7 agonist on tumor size and cytokine levels
NCT04576429	Melanoma	Effect of ICIs on PFS and a variety of cytokines
NCT03854032	Squamous cell carcinoma	Effect of immunotherapy on OR, immune cell polarization, and inflammatory markers
NCT04698213	Metastatic renal carcinoma	Effect of immunotherapy on ORR and cytokines
NCT04116138	Glioblastoma	Safety and feasibility of Salovum and its effect on inflammatory cytokine levels
NCT04135079	Multiple myeloma	Immune transcriptome profile, immune signatures, and cytokine profiles
NCT03475628	Multiple myeloma	Effect of daratumumab on bone formation and resorption markers
NCT00398515	Multiple myeloma	Max tolerated dosage and side effects of lenalidomide and temsirolimus, including their effect on serum cytokines
NCT01329289	Multiple myeloma	Effect of pasireotide LAR on CR, PR, cytokine levels, and pathways
NCT02471820	Multiple myeloma	Efficacy and safety of lenalidomide and its effect on PFS and cytokine levels
NCT03392584	Rectal	Effect of abdominoperineal resection on metabolic and inflammatory parameters
NCT03196180	Cervical intraepithelial neoplasia and cervical squamous cell carcinoma	Side effects of fluorouracil and imiquimod and changes in the expression of biomarkers of local immune activation
NCT03873805	Castration-resistant prostate carcinoma and metastatic prostate carcinoma	Effect of CAR T cells on OS, PFS, and serum cytokine profile
NCT04177810	Metastatic pancreatic carcinoma	Evaluate safety and clinical activity of plerixafor (anti-CXCR4) in combination with cemiplimab (anti-PD-1 antibody)

Abbreviations: CAR, chimeric antigen receptor; CCL3/MIP-1α, C-C motif chemokine ligand 3/macrophage inflammatory protein 1α; CR, complete response; CXCR4, C-X-C motif chemokine receptor 4; ICI, immune checkpoint inhibitors; LAR, long-acting-release; OR, objective response; ORR, overall response rate; OS, overall survival; PD-1, programmed cell death protein 1; PFS, progression-free survival; PR, partial response; TLR, Toll-like receptor.

**Table 3:** Ongoing clinical trials focusing on CRP as a biomarker

Clinical trial identifier	Cancer type	Purpose of study
NCT04366713	Breast	Effect of neratinib on colon pathology Changes in CRP to be measured as a secondary outcome
NCT01472094	Breast	Predict chemotherapy toxicity and assess potential biomarkers
NCT04205786	Breast	Effect of vitamin B_12_ on joint pain and associated inflammatory cytokines
NCT03748030	Breast	Impact of radiotherapy on cardiac inflammation
NCT04361240	Breast	Impact of radiotherapy on cardiotoxicity
NCT03872388	Breast	Impact of adding atorvastatin to neoadjuvant chemotherapy
NCT03330847	Breast	Examine efficacy and safety of olaparib on survival and associated parameters
NCT01693783	Cervical	Examine efficacy of ipilimumab on response, survival, and associated parameters
NCT02713386	Ovarian	Study side effects and optimum dosage of chemotherapy as well as any survival benefits
NCT03919461	Colorectal	Effect of propranolol and etodolac on disease free survival and biomarkers
NCT01105169	Colorectal	Impact of dietary supplements on various biomarkers and related proteins
NCT04149613	Colorectal	Determine prognostic value of inflammatory markers and microRNA
NCT03559335	Colorectal	Examine various inflammatory biomarkers in postoperative complications
NCT03798626	Colorectal, gastroesophageal, and renal	Examine efficacy of gevokizumab in combination with the standard of care therapy
NCT04324567	Rectal	Impact of surgery on CRP levels and survival
NCT04819958	Gastric	Effect of immunological heterogeneity on survival rate and CRP
NCT02792881	Gastric	Effect of surgery on morbidity, survival, and biomarkers
NCT03645317	Lung	Impact of radiotherapy on various blood parameters
NCT04305613	Lung	Effect of chemoradiation on cytokine levels, survival, and cardiac stress
NCT03300817	Lung	Study immunogenicity and efficacy of MUC1 vaccine
NCT04303975	Nasopharyngeal	Explore the association of CRP and radiotherapy
NCT04617756	Urothelial	Safety and efficacy of durvalumab plus neoadjuvant chemotherapy
NCT04183478	Pancreatic	Study the efficacy and safety of a peptidoglycan, and its impact on survival and blood parameters
NCT03447314	Solid tumors	Study optimum dosage and efficacy of a TLR4 agonist in combination with immunotherapies

Abbreviations: CRP, C-reactive protein; MUC1, mucin 1; TLR, Toll-like receptor.

## A SIMPLIFIED EQUATION DESCRIBING RANDOM CELL MIGRATION AND CHEMOTAXIS

For cells tracked in the single plane of focus of a microscope lens in vitro or in vivo, the mean-squared displacement (MSD) of an individual migratory cell can be simplified as follows:

$$\langle \Delta r^{2}\;(t)\rangle ={\langle \Delta r^{2}\;(t)\rangle }_{x}+{\langle \Delta r^{2}\;(t)\rangle }_{y}\;\;\approx v^{2}t^{2}+4Dt.$$

The MSD consists of both chemotaxis of speed *v* along a chemotactic axis *x* (which has units of length per unit time), corresponding to the direction of the chemotactic gradient, and random basal migration, characterized by the diffusion parameter *D* (which has units of length squared per unit time). For a cell undergoing both chemotaxis and random migration, random migration will dominate at short timescales:

$$\langle \Delta r^{2}\;(t)\rangle ≅4Dt,\;\text{for}\;t\ll 4D∕v^{2}.$$

In contrast, chemotaxis will dominate at long timescales:

$$\langle \Delta r^{2}\;(t)\rangle ≅v^{2}t^{2},\;\text{for}\;t\gg 4D∕v^{2}.$$

Between these two temporal extremes, trajectories of cells will be a so-called biased random walk, combining a unidirectional straight line and random walk migration (42, 73, 74).

## DISTRIBUTION OF IMMUNE AND CANCER CELL MOVEMENTS IN THE ABSENCE OF CHEMOATTRACTANTS

In the mouse brain infected with the pathogen *Toxoplasma gondii*, tracking of cells using two-photon microscopy indicates that CD8^+^ T cells undergo random migration that follows generalized Lévy walks instead of Brownian walks. Therefore, the distribution of movements of these cells is not Gaussian:

$$P(\Delta r)∼e^{-\frac{\Delta r^{2}}{2}}.$$

Rather, it follows the form

$$P(\Delta r)∼\left(\frac{1}{\Delta r^{\frac{3}{2}}}\right)e^{-\frac{1}{2\Delta r^{2}}}.$$

This distribution acknowledges that T cells display small movements interspaced with long exploratory excursions (75, 76). Such Lévy flights allow for efficient target searching.

In the absence of immune cells, cancer cells undergo anisotropic random walks characterized by main and secondary axes of migration parameterized by two persistence times and two diffusion coefficients. The distribution of movements follows an exponential,

$$P(\Delta r)∼e^{-\frac{\Delta r}{2}},$$

due to large cell-to-cell variations. In vivo, the average speed of T cells is in the range of 1–10 μm/min, while that of cancer cells is much slower, typically less than 1 μm/min.

## Abbreviations

FAK: focal adhesion kinase
TME: tumor microenvironment
EV: extracellular vesicle
MDSC: myeloid-derived suppressor cell
Treg: T regulatory cell
NK: natural killer
DC: dendritic cell
TAM: tumor-associated macrophage
IL: interleukin
PD-1: programmed cell death protein 1
CCL: C-C motif chemokine ligand
TNBC: triple-negative breast cancer
CXCL: C-X-C motif chemokine ligand
CXCR: CXC chemokine receptor
CCR: CC chemokine receptor
SDF-1: stromal cell-derived factor 1
EMT: epithelial-to-mesenchymal transition
TGF-β: transforming growth factor β
GM-CSF: granulocyte-macrophage colony-stimulating factor
ECM: extracellular matrix
MMP: matrix metalloproteinase
TEB: tumor-educated B cell
TNF: tumor necrosis factor
TRAIL: TNF-related apoptosis–inducing ligand
WASP: Wiskott–Aldrich syndrome protein
ROS: reactive oxygen species
NO: nitric oxide
MIP: macrophage inflammatory protein
CRP: C-reactive protein
